# Awareness of risk factors for cancer: a comparative study of Sweden and Denmark

**DOI:** 10.1186/s12889-015-2512-9

**Published:** 2015-11-23

**Authors:** Magdalena Lagerlund, Line Hvidberg, Senada Hajdarevic, Anette Fischer Pedersen, Sara Runesdotter, Peter Vedsted, Carol Tishelman

**Affiliations:** Department of Learning, Informatics, Management and Ethics (LIME), Karolinska Institutet, Stockholm, SE 171 77 Sweden; Research Centre for Cancer Diagnosis in Primary Care (CaP), Research Unit for General Practice, Department of Public Health, Aarhus University, Aarhus, Denmark; Section for General Medical Practice, Department of Public Health, Aarhus University, Aarhus, Denmark; Department of Nursing, Umeå University, Umeå, Sweden; Innovation Centre, Karolinska University Hospital, Stockholm, Sweden

**Keywords:** Risk factors, Cancer, Awareness, Denmark, Sweden

## Abstract

**Background:**

Sweden and Denmark are neighbouring countries with similarities in culture, healthcare, and economics, yet notable differences in cancer statistics. A crucial component of primary prevention is high awareness of risk factors in the general public. We aimed to determine and compare awareness of risk factors for cancer between a Danish and a Swedish population sample, and to examine whether there are differences in awareness across age groups.

**Methods:**

Data derive from Module 2 of the International Cancer Benchmarking Partnership. Telephone interviews were conducted with 3000 adults in Denmark and 3070 in Sweden using the Awareness and Beliefs about Cancer measure. Data reported here relate to awareness of 13 prompted risk factors for cancer. Prevalence ratios with 95 % confidence intervals were calculated to examine associations between country, age, and awareness of risk factors.

**Results:**

Over 90 % of respondents in both countries recognized smoking, use of sunbeds and ionizing radiation as risk factors for cancer. Lowest awareness (<50 %) was found for HPV-infection, low fruit and vegetable intake and alcohol intake. Swedish respondents reported higher awareness than Danish respondents for ten of the 13 risk factors studied. Respondents from Denmark reported higher awareness only regarding low fruit and vegetable intake and use of sunbeds. Low physical activity was the only risk factor for which there was no difference in awareness between the countries. A decline in awareness was generally seen with increasing age in both countries, but deviating patterns were seen for alcohol intake, red/processed meat, obesity and age 70+.

**Conclusions:**

This study supports findings from other European studies that generally demonstrate modest public awareness of many established cancer risk factors. Efforts should be made to improve awareness of the cancer risk factors HPV-infection, low fruit and vegetable intake and alcohol intake, which showed particularly low awareness in both countries. Previous studies indicate that repeated, broad campaigns are successful, and suggest that a multimedia approach is used.

## Background

Recent statistics show that cancer was the second most common cause of death in the European Union in 2011 [1] and that important international differences exist in cancer survival [2]. The International Cancer Benchmarking Partnership (ICBP) was initiated with the purpose of investigating international differences in cancer survival and their possible causes, and includes jurisdictions in Australia, Canada, Denmark, England, Northern Ireland, Norway, Sweden and Wales [3]. In the second of five ICBP modules, cancer awareness and beliefs in the general population were investigated.

In the present study based on data from ICBP Module 2, we focus on Denmark and Sweden, two Nordic countries with many similarities in culture, healthcare systems, and economic status, but with variations in cancer statistics. World age-standardized mortality rates from cancer (all sites) in 2013 were considerably lower in Sweden (102 (male) and 84 (female) per 100 000) than in Denmark (138 (male) and 109 (female) per 100 000) [4]. Denmark even had the highest estimated cancer incidence among 184 countries worldwide in 2012, while Sweden ranked 24 in incidence [5].

It is widely accepted that awareness is an important condition, although not sufficient on its own, for stimulating behavioural change. For some cancers, risk can be reduced through behavioural changes of modifiable cancer risk factors. Furthermore, in the case of both modifiable and non-modifiable factors, awareness might promote appropriate health-seeking behaviour. By addressing known modifiable risk factors it has been estimated that a third to half of all cancers in the developed world could be prevented, and that early diagnoses and effective treatments could cure another one third [6–8]. Assessing awareness of risk factors for cancer among the general public is thus an important step in identifying potential areas where awareness may need to be raised.

Awareness of cancer risk factors has not been extensively examined in Europe apart from a few UK [9–12] and multi-country studies [13, 14], with one previous multi-country study finding that adults in Denmark had lower awareness of colorectal cancer risk factors than adults in Sweden [14], although no explanation for this was presented. For many established risk factors for cancer, awareness levels have been found to be low [9, 10, 12, 14–16], but vary widely depending on what countries, cancer forms and risk factors were investigated, as well as study characteristics. Broad, updated, and context specific knowledge of awareness levels is needed to support planning and implementation of appropriate interventions.

Successful implementation of risk factor awareness campaigns may also be facilitated by knowledge of which subgroups of the population might benefit most from specific campaigns. An association between age and awareness of established risk factors for cancers has been indicated in some studies [9, 10, 15, 17, 18]; however no consistent age-related pattern emerges across different cancers and risk factors, and other studies find no effect of age [11, 19–21]. Even though people over age 70 are at highest risk of developing cancer it is important to also gauge information needs, raise awareness and encourage healthier lifestyles in younger people. Both Denmark and Sweden collected ICBP Module 2 data from ages 30 and older, enabling an investigation of awareness across a wide age span, to provide a basis for future public health initiatives.

The aim of this study is therefore to determine and compare awareness of a number of established risk factors for cancer between a Danish and a Swedish population sample, and to examine whether there is a difference in awareness of risk factors across age groups in Denmark and Sweden.

## Methods

### Study population and data collection

We used data collected through a telephone survey for the ICBP Module 2. The target sample size was 1000 respondents aged 30-49 years and 2000 respondents aged ≥50 years in each country. Using simple random sampling a total of 20,000 residents 30-49 years of age and 40,000 residents aged 50 and older were selected from the Danish Civil Registration System (CRS). In Sweden, a total of 8000 residents 30-49 years of age and 15,000 residents aged 50 and over were selected from the Swedish Population and Address Register (SPAR) for the Uppsala-Örebro and Stockholm-Gotland healthcare regions. Names and/or addresses as listed in the CRS and SPAR were supplemented with landline and/or mobile phone numbers by national market research and consulting firms (NN Markedsdata in Denmark and Infodata in Sweden). The survey was conducted from May 31 to July 4, 2011 in Denmark and from August 15 to September 30, 2011 in Sweden. Computer-assisted telephone interviews were carried out by trained native-speaking interviewers from the research company Ipsos MORI (for further details about the methodological procedures, see references [22, 23]).

Table 1 shows the data collection process to obtain the final number of 3000 respondents in Denmark and 3070 respondents in Sweden. Interviews were only completed with respondents who were able to understand and speak Danish or Swedish, respectively. A response rate of 31 % in Denmark and 27 % in Sweden was achieved, estimated as the number of completed interviews divided by the number of persons eligible. Among those successfully contacted after up to seven efforts (8046 in Denmark and 8121 in Sweden) interviews were completed for 37 % and 38 %, respectively.

**Table 1:** Data collection process and survey response

	Denmark	Sweden
Initial study base	60,000	23,000
Persons with research protection	6570	NA
Persons with no obtainable phone number	6309	3958
Further exclusions^a^	55	NA
Eligible for being contacted	47,066	19,042
Total number of persons approached	11,297	12,750
Technical annulment	1664	1113
Number of ineligible persons	33	102
Number of persons eligible	9600	11,535
Number of persons who could not be contacted after seven attempts	1554	3414
Number of persons eligible and made contact to	8046	8121
Refused or did not complete the interview	5046	5051
Completed interviews	3000	3070

*NA* Not applicable

^a^Before start of data collection in Denmark, it was checked with CRS whether the persons 1) had a newly established research protection status (n = 7), 2) had emigrated from Denmark (n = 11) or 3) had passed away (n = 37)

### Survey measure and study variables

Module 2 data was aimed to measure cancer awareness and beliefs in the general population using the Awareness and Beliefs about Cancer (ABC) instrument (for further information on instrument development and testing, please see references [22, 23]). This instrument consists of a core section covering questions on awareness and beliefs about cancer, and perceived barriers to healthcare seeking, and optional sections on awareness of risk factors for cancer and on cancer screening beliefs and behaviours. In addition, information about sociodemographic factors, smoking status, self-rated health and personal experience of cancer (self and/or other, if any) was collected.

The analyses reported here relate to awareness of the following 13 risk factors for cancer: smoking, second-hand smoke, drinking more than 1 unit of alcohol a day, low fruit and vegetable intake, red/processed meat, obesity, sunburn in childhood, being over 70 years of age, having a close relative with cancer, HPV-infection, low physical activity, use of sunbeds, and exposure to ionizing radiation. These questions constituted an optional survey component that both Sweden and Denmark included. Using a recognition method, the respondents were presented with each of the above risk factors after the following instruction: ‘I am now going to read out a list of things which may or may not increase your chances of getting cancer. For each one can you tell me how much you agree or disagree that it may increase your chances of getting cancer?’. The response options were dichotomized into *awareness* (tend to agree and strongly agree) and *lack of awareness* (tend to disagree, strongly disagree, and don’t know). ‘Don’t know’ was included in the category ‘lack of awareness’ because these respondents were not aware that the factor in question was a risk factor for cancer, hence lacked awareness. The proportion of respondents answering ‘don’t know’ was below 4 % for all risk factors except *red/processed meat* (6.4 %) and *HPV-infection* where 62 % responded they didn’t know what HPV is and 1.6 % answered ‘don’t know’. All missing observations (no answer) for risk factors were excluded from analysis. A composite measure of awareness was also created by counting the number of known risk factors for each respondent. Data is further presented in groups based on country (Denmark/Sweden), and 5-year age groups (30-34, 35-39… to 80+) or 10-year age groups (30-39, 40-49… to 70+).

### Statistical analysis

Descriptive characteristics are reported as mean (standard deviation (SD)) or count (percent) to describe awareness of risk factors among Danish and Swedish respondents and across age groups. In a multivariable analysis (generalised linear model, GLM) using prevalence ratios (PRs) with 95 % confidence intervals (CIs), we estimated the association between country and awareness of each of the 13 risk factors when adjusting for age (continuous), gender (men; women), country of birth (Denmark/Sweden; other), cohabitation (living with partner; living alone), education (primary/lower secondary school; upper secondary; bachelor and higher), and experience of cancer (self and/or family/friends vs. none). Furthermore, interaction terms were included to test effect modification by age group (10-year intervals). Data were analysed using SAS 9.3, and IBM SPSS Statistics 20.

### Ethics approval

The Danish study was approved by the Danish Data Protection Agency (J. no. 2011-41-6237) and the Danish Health and Medicines Authority. In accordance with the Central Denmark Region Committees on Biomedical Research Ethics the study needed no further approval (Report no. 128/2010). The Swedish study was approved by the research ethics committee at Karolinska Institutet (Ref. no. 2011/699-31/2).

## Results

The sociodemographic characteristics of the Danish and Swedish samples are presented in Table 2. Some statistically significant differences were noted between the samples. In Sweden the average age of respondents was slightly higher than in Denmark, and a higher proportion indicated that they lived alone, had high education, were born abroad, and had no close experience of cancer.

**Table 2:** Sociodemographic characteristics of the respective samples in Denmark (n = 3000) and Sweden (n = 3070)

Sociodemographic characteristic	Denmark	Sweden
	n (%)	n (%)
Gender		
Females	1659 (55.3)	1718 (56.0)
Males	1341 (44.7)	1352 (44.0)
Age groups		
30-39	416 (13.9)	445 (14.5)
40-49	584 (19.5)	586 (19.1)
50-59	746 (24.9)	633 (20.6)
60-69	764 (25.5)	836 (27.2)
70+	490 (16.3)	570 (18.6)
Age, mean (SD)*	55.9 (13.3)	56.6 (14.1)
Cohabitation***		
Living with a partner	2354 (78.5)	2272 (74.1)
Living alone	644 (21.5)	793 (25.9)
Missing	2	5
Education		
Primary and lower secondary	565 (18.9)	563 (18.4)
Upper secondary	1400 (46.9)	1249 (40.8)
Bachelor and PhD^a^	1020 (34.2)	1248 (40.8)
Missing	15	10
Country of birth***		
Country of current residence (DK/SE)	2854 (95.1)	2687 (87.6)
Other	146 (4.9)	381 (12.4)
Missing	0	2
Experience of cancer (self and/or family/friend)**		
Yes	2526 (84.3)	2496 (81.4)
No	472 (15.7)	572 (18.6)
Missing	2	2

*p < 0.05, ** < 0.01, *** *p* < 0.001, between countries

^a^Difference between the highest level and the two lower levels is statistically significant (*p* < 0.001)

Table 3 presents frequencies and prevalence ratios (crude and adjusted) of lack of awareness of individual risk factors by country. The Danish respondents were *more* likely to demonstrate a lack of awareness of ten of the 13 risk factors (smoking, second-hand smoke, alcohol intake, red/processed meat, obesity, sunburn in childhood, age over 70 years, having a close relative with cancer, HPV-infection, and ionizing radiation) compared to the Swedish respondents but they were *less* likely to lack awareness of low fruit and vegetable intake and use of sunbeds being risk factors for cancer. Reported awareness of low physical activity did not differ significantly between the Danish and Swedish respondents. The largest difference in reported lack of awareness between Denmark and Sweden were seen for the risk factors age over 70 years (49 % vs. 34 %), HPV-infection (76 % vs. 64 %), and having a close relative with cancer (30 % vs. 18 %).

**Table 3:** Prevalence of lack of awareness of individual risk factors for Denmark versus Sweden

Risk factor	N		%		Crude	Adjusted^b^
	Denmark	Sweden	Denmark	Sweden	PR (95 % CI)^a^	PR (95 % CI)^a^
Smoking	104/2998	50/3070	3.5	1.6	2.13 (1.53-2.97)	2.35 (1.68-3.30)
Second-hand smoke	368/2999	174/3070	12.3	5.7	2.17 (1.82-2.57)	2.31 (1.94-2.76)
Alcohol intake	1700/3000	1613/3069	56.7	52.6	1.08 (1.03-1.13)	1.08 (1.03-1.13)
Low fruit and vegetable intake	1769/3000	1993/3065	59.0	65.0	0.91 (0.87-0.94)	0.90 (0.87-0.94)
Red/processed meat	1512/2999	1271/3066	50.4	41.5	1.22 (1.15-1.29)	1.21 (1.15-1.28)
Obesity	996/2999	916/3070	33.2	29.8	1.11 (1.03-1.20)	1.10 (1.02-1.18)
Sunburn in childhood	1052/2999	909/3068	35.1	29.6	1.18 (1.10-1.27)	1.19 (1.11-1.28)
>70 years of age	1481/2999	1057/3070	49.4	34.4	1.43 (1.35-1.52)	1.41 (1.33-1.50)
Close relative with cancer	893/2998	541/3069	29.8	17.6	1.69 (1.54-1.86)	1.71 (1.56-1.88)
HPV-infection	2291/3000	1978/3069	76.4	64.5	1.18 (1.15-1.22)	1.16(1.12-1.20)
Low physical activity	1063/3000	1127/3069	35.4	36.7	0.96 (0.90-1.03)	0.98 (0.91-1.05)
Use of sunbeds	136/3000	204/3068	4.5	6.6	0.68 (0.55-0.84)	0.74 (0.60-0.92)
Ionizing radiation	305/2998	191/3069	10.2	6.2	1.63 (1.37-1.94)	1.68 (1.41-2.01)

^a^Prevalence ratios (PRs) and 95 % confidence intervals (CIs) with Denmark compared to Sweden

^b^Adjusted for gender, age, cohabitation, education, country of birth and experience of cancer

**Fig. 1:**
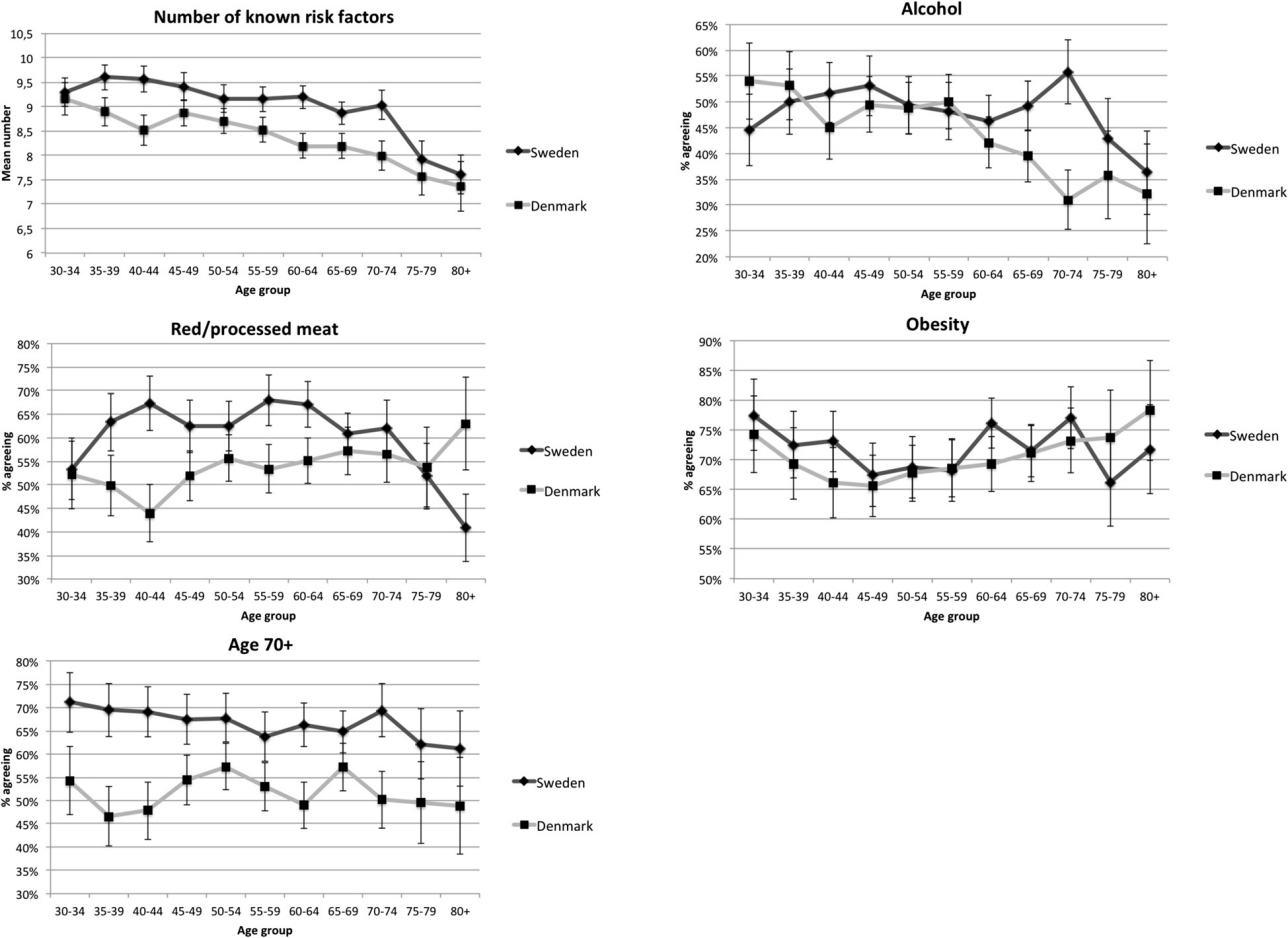
Awareness of risk factors for cancer by age group. Point estimates with 95 % confidence intervals

A downward trend in awareness with increasing age was observed in both countries, both in general, represented by the mean number of risk factors that were known (Fig. 1), and for most individual risk factors (data not shown). Exceptions to this pattern were found for awareness of alcohol intake, red/processed meat, obesity and age over 70 years (Fig. 1).

When examining whether age group modified the effect of country on awareness of each individual risk factor, we found statistically significant interaction effects for the risk factors alcohol intake, red/processed meat and having a close relative with cancer (Table 4). The association between country and awareness of alcohol intake as a risk factor was only statistically significant in the age groups from 60 and older; awareness of the risk factor red/processed meat was associated with country in all age groups but the oldest (70+); and awareness of the risk factor ‘having a close relative with cancer’ was most strongly associated with country among those 40-69 years of age. Furthermore, there was more effect of country among younger age groups compared to those 70+ for awareness of smoking and ionizing radiation. In all these instances the effect of country was such that respondents from Denmark were more likely to lack awareness than respondents from Sweden.

**Table 4:** Lack of awareness of risk factors for Denmark vs. Sweden. Interaction and stratification by age

Risk factor	Interaction	PR (95 % CI)^a^				
	Country* age					
		Age 30-39	Age 40-49	Age 50-59	Age 60-69	Age 70+
	*p*-value					
Smoking	0.973	--^b^	--^b^	2.37 (0.96-5.84)	2.47 (1.39-4.39)	1.73 (1.00-3.01)
Second-hand smoke	0.602	--^b^	--^b^	2.39 (1.61-3.54)	2.19 (1.61-2.97)	2.36 (1.71-3.25)
Alcohol intake	<0.001	0.91 (0.79-1.04)	1.08 (0.96-1.21)	0.97 (0.87-1.07)	1.12 (1.03-1.23)	1.27 (1.15-1.39)
Low fruit and vegetable intake	0.347	0.93 (0.84-1.04)	0.95 (0.86-1.05)	0.84 (0.77-0.91)	0.94 (0.87-1.01)	0.87 (0.80-0.95)
Red/processed meat	<0.001	1.25 (1.08-1.44)	1.43 (1.26-1.63)	1.25 (1.10-1.42)	1.22 (1.09-1.37)	0.98 (0.86-1.11)
Obesity	0.442	1.19 (0.96-1.47)	1.18 (1.00-1.39)	0.99 (0.85-1.16)	1.12 (0.97-1.30)	0.99 (0.82-1.19)
Sunburn in childhood	0.966	1.15 (0.92-1.42)	1.26 (1.04-1.52)	1.14 (0.98-1.34)	1.22 (1.06-1.40)	1.14 (0.98-1.33)
>70 years of age	0.119	1.66 (1.41-1.95)	1.49 (1.30-1.72)	1.28 (1.12-1.46)	1.35 (1.20-1.52)	1.44 (1.26-1.65)
Close relative with cancer	0.010	1.33 (0.97-1.82)	2.02 (1.53-2.67)	1.84 (1.48-2.28)	2.01 (1.67-2.42)	1.38 (1.18-1.62)
HPV-infection	0.204	1.12 (1.01-1.25)	1.16 (1.07-1.26)	1.23 (1.14-1.32)	1.19 (1.12-1.26)	1.11 (1.05-1.18)
Low physical activity	0.181	0.82 (0.66-1.01)	1.04 (0.88-1.21)	0.88 (0.76-1.01)	1.03 (0.90-1.17)	1.02 (0.88-1.18)
Use of sunbeds	0.927	0.81 (0.44-1.51)	0.75 (0.42-1.34)	0.87 (0.55-1.39)	0.60 (0.39-0.92)	0.70 (0.46-1.06)
Ionizing radiation	0.226	--^b^	1.65 (1.07-2.57)	2.17 (1.31-3.60)	1.85 (1.31-2.59)	1.30 (0.96-1.76)

^a^Prevalence ratios (PRs) and 95 % confidence intervals (CIs) adjusted for gender, cohabitation, education, country of birth and experience of cancer

^b^Model could not converge due to small cell-sizes

## Discussion

In this population-based study among adults aged 30 years or older in Denmark and Sweden the highest level of cancer risk factor awareness was reported for smoking, use of sunbeds and ionizing radiation with awareness levels of over 90 %. In both countries, the lowest levels of awareness were found for HPV-infection, low fruit and vegetable intake and alcohol intake as risk factors for cancer. Swedish respondents reported higher risk factor awareness than Danish respondents for ten of 13 risk factors studied. A decline in awareness was generally seen with increasing age in both countries, although deviating patterns were seen for alcohol intake, red/processed meat, obesity and age 70+.

Most of the risk factors examined are modifiable by the individual, whereas having a close relative with cancer and age over 70 years are not modifiable, and ionizing radiation and second-hand smoke are variable, and arguably under less individual control. We saw no clear pattern with respect to level of awareness among modifiable and non-modifiable risk factors. However, the risk factors for which Danish respondents reported higher awareness were both modifiable (low fruit and vegetable intake and use of sunbeds). The risk factors where awareness differed most between Denmark and Sweden, with higher awareness among Swedish respondents, were primarily non-modifiable (including age over 70 years, having a close relative with cancer and HPV-infection). A previous European study also found that awareness of family history and older age as risk factors specifically for colorectal cancer was higher in Sweden compared to Denmark [14]. Similarly, Forbes et al.’s initial ICBP Module 2 study, restricted to respondents ≥50 years, found higher awareness in Sweden (38 %) than in Denmark (25 %) that cancer risk is higher in people aged ≥70 years than in younger age groups [22]. Considering the importance of age as a risk factor for most cancers and the ageing populations the awareness of this risk factor was notably low among the respondents in our study, especially in Denmark.

Exposure to HPV-infections can to some extent be modified through sexual behaviour [24] and vaccinations. Considering the large amount of publicity given to HPV-vaccinations in both Denmark and Sweden, the low awareness of HPV-infections as a risk factor for cancer is also noteworthy, with as much as 62 % of respondents reporting not knowing what HPV is. Similar rates were found among 18-45-year old women in a Nordic study conducted in 2005, just before the release of HPV-vaccines [25].

It is unclear how to explain that the respondents in Sweden generally demonstrated higher awareness. Possibly this reflects an effect of different welfare policies or cultural differences. Management research shows large similarities between cultures in Sweden and Denmark, but a tendency for Swedish culture to be somewhat more collective and to have a stronger inclination to avoid uncertainty [26, 27]. Furthermore, Vallgarda [28] suggest that whereas the Swedish welfare policy programmes stress political responsibility to improve population health, the Danish programme is more liberal, emphasizing individual responsibility and autonomy. Examples of this are the alcohol retail monopoly that exists in Sweden but not in Denmark and differences in cancer screening programs, with nation-wide mammography screening established earlier in Sweden (1997) [29] than in Denmark (2008) [30].

The relatively low response rates may lead to an over-estimation of public awareness in both countries due to selection bias. Since the response rate was lower in Sweden there may be a more pronounced over-estimation of awareness, which would risk exaggerating the difference between countries. Immigrants and men 30-49 year of age were similarly underrepresented among the Swedish and Danish respondents. However, there was a higher proportion of people with higher education in the Swedish sample when comparing the Swedish and Danish respondents to the age-specific populations in their respective countries. A partial explanation for this may be that the two Swedish healthcare regions chosen for this study have a higher average level of education compared to the country as a whole. Hvidberg et al. [18] has found that low education was associated with lower awareness of risk factors for cancer among the Danish ICBP-respondents. To diminish the potential confounding effect that such selection mechanisms may have on the outcome we adjusted for education, age and other sociodemographic factors that we had data for in the multivariate analysis.

The questions used for this study were not particularly sensitive but we still acknowledge that there may have been a tendency among respondents to give socially desirable answers, which could have led to an underestimation of the lack of awareness. However, since the same questions and data collection methods were used in Sweden and Denmark there is little reason to believe that this type of information bias would have affected comparisons between countries.

Awareness of sunbed use as a risk factor for cancer was higher in Denmark, which may be a result of the Danish anti-sunbed campaign, specifically targeting young people. This campaign gave rise to an intense public debate about sunbed use, and subsequent use of sunbeds among the youngest age groups decreased [31]. Other Danish nationwide campaigns have been addressing fruit and vegetable consumption, physical activity and obesity prior to data collection for the current study [32]. Also throughout Sweden an annual campaign week concerning physical activity and healthy eating habits has been organized since 2010. However, it is important to point out that the cross-sectional design of the current study does not permit drawing conclusions about the impact of health campaigns or changes in awareness over time. Furthermore, even though high awareness of risk factors is an important component of primary prevention of cancer, it is not a straightforward predictor of behaviour [33, 34], which is the more important determinant of cancer outcomes.

The decrease in awareness with age may be an effect of the use of a prompted question format. Waller et al. [35] found that this format resulted in significantly higher awareness of risk factors and warning signs of cancer compared with the unprompted format, but decreasingly so with increasing age. Another possibility may be that younger age groups are more readily reached by health messages in different media and/or are more prone to assimilate new information that may perhaps challenge previously held beliefs. Interestingly, the youngest (30-34 years) age groups did not differ so much by country in terms of number of known risk factors. It may be possible that recent campaigns in Denmark have succeeded in reaching a younger segment of the population, or there may be an internationalization of information via e.g. social media and the Internet leading to decreased differences particularly among younger age groups. In line with our study, Hawkins et al. [17] found that 35-64 year olds in the U.S. were able to cite more cancer prevention strategies for cancer in general, compared to older age groups. A UK study found age-differences in awareness of infections, alcohol intake, sunburn and having a relative with cancer, but less of a consistent pattern [10].

Despite finding some interaction effects, the differences in awareness between Sweden and Denmark were generally consistent across age groups. The stratified analyses showed that the difference in awareness of alcohol intake as a risk factor was only statistically significant among those aged 60 and over. It appears that there may be a cohort effect of people who were teenagers and young adults in the 60s and 70s and who may have experienced different cultures around alcohol in Sweden and Denmark.

Even though the large population-based samples for both countries was one of the strengths of the present study, we still were challenged by smaller cell-sizes for some risk factors with low variability in responses in the age-stratified analyses. This same issue also caused a lack of discrimination in analysis of data from older respondents (70+), which is regrettable given the aging population and the potentially interesting patterns in the data from this group.

## Conclusions

This study supports findings from other European studies that generally demonstrate modest public awareness of many established cancer risk factors. We found that Swedish respondents reported higher awareness than Danish respondents for ten of 13 cancer risk factors studied. A decline in awareness was generally seen with increasing age in both countries. Efforts can be made to increase awareness of the cancer risk factors HPV-infection, low fruit and vegetable intake and alcohol intake, which both the Swedish and the Danish population showed particularly low awareness of. Also, considering how strong increasing age is as a risk factor for cancer, it might be important to further increase awareness thereof as a means to stimulate appropriate healthcare seeking behaviour, especially in the Danish population.

### Implications

One way to increase awareness, which has shown some success, could be to deliver tailored multiple risk factor health and lifestyle advice in conjunction with existing screening programs [36]. Previous studies indicate that repeated, broad campaigns, as well as a multimedia approach, including e.g. television and the Internet, to reach different socioeconomic subgroups, are needed to attain changes in attitudes and behaviours [37–39].
